# A comparative study of biomechanical assessments in laboratory and field settings for manual material handling tasks using extractor tools and exoskeletons

**DOI:** 10.3389/fbioe.2024.1358670

**Published:** 2024-05-20

**Authors:** Maryam Shakourisalim, Xun Wang, Karla Beltran Martinez, Ali Golabchi, Sarah Krell, Mahdi Tavakoli, Hossein Rouhani

**Affiliations:** ^1^ Department of Mechanical Engineering, University of Alberta, Edmonton, AB, Canada; ^2^ Department of Civil and Environmental Engineering, University of Alberta, Edmonton, AB, Canada; ^3^ EWI Works Inc., Edmonton, AB, Canada; ^4^ EPCOR Utilities Inc., Edmonton, AB, Canada; ^5^ Department of Electrical and Computer Engineering, University of Alberta, Edmonton, AB, Canada; ^6^ Glenrose Rehabilitation Hospital, Edmonton, AB, Canada

**Keywords:** low back pain, ergonomic risk assessment, inertial measurement unit, occupational exoskeleton, electromyography

## Abstract

To enhance physical capabilities of workers who regularly perform physically demanding tasks involving heavy lifting and awkward postures, various tools and occupational exoskeletons can be used. Most of the studies aiming to explore the efficiency of these tools and exoskeletons have been performed in confined and controlled laboratory spaces, which do not represent the real-world work environment. This study aimed to compare the outcome of biomechanical assessment of using a back support exoskeleton and assistive tools (Lever and Jake) in the procedure of a high demanding manual material handling task versus the results found by performing the same task in a laboratory. Ten able-bodied participants and ten able-bodied utility workers performed the same manhole removal task in-lab and in-field, respectively, with the aid of an exoskeleton and Lever and Jake tools. Muscle activity and Rapid Entire Body Assessment (REBA) scores were recorded using surface electromyography and inertial measurement units, respectively and compared between in-lab and in-field trials. The field experiments indicated significant differences (*p* < 0.05) in normalized muscle activity across most muscles when compared to laboratory data. These results revealed how muscle activity is affected by the controlled lab setting compared to real-world field conditions. However, REBA scores indicate similar ergonomic implications regardless of the utilization of exoskeletons or tools. These findings underscore that real-world field assessments are crucial for evaluating ergonomic risks and effects of occupational exoskeletons and tools to account for environmental factors and workers’ skills in ergonomic evaluations of this nature.

## 1 Introduction

To reduce the prevalence of work-related musculoskeletal disorders (WMSDs), employers are increasingly investing in equipment, tools, and training initiatives designed to minimize the physical strain associated with physically demanding tasks, and subsequently improve the wellbeing of the workers, enhance workplace productivity, and reduce the economic burden of injuries (Coenen et al., 2014). For example, occupational exoskeletons are proposed to reduce the risk of fatigue and chronic WMSDs. Exoskeletons are wearable devices constructed from lightweight materials and integrate mechanical components to enhance the physical capabilities of workers, addressing challenges like lifting heavy objects, performing repetitive tasks, or enduring extended periods of standing or kneeling (Kim et al., 2019; Andrade and Nathan-Roberts, 2022).

Numerous studies have focused on ergonomic risk analysis and the effect of physical assistance devices, such as occupational exoskeletons. However, these studies are mostly conducted in laboratory settings (Baltrusch et al., 2019; Theurel and Desbrosses, 2019; De Bock et al., 2022). Laboratory assessments provide controlled environments for detailed biomechanical and physiological measurements, isolating the exoskeleton’s effects from other variables and allow for precise and repeatable measurements (Baltrusch et al., 2019). These controlled conditions may not accurately represent the complexities and variability of real-world tasks, which can limit the generalizability of findings and impact ecological validity of assessments (Hoogendoorn et al., 2000; Wami et al., 2019). In contrast, field evaluations conducted in actual work environments allow researchers to observe and evaluate workers in their natural settings, which ensures that the assessments are contextually relevant and offer crucial insights into the exoskeleton’s real-world performance, usability, and user acceptance (De Bock et al., 2020; Gull et al., 2020; Bennett et al., 2023). In-field ergonomic risk assessments are also subject to limitations such as the influence of uncontrolled environmental variables and limited measuring equipment (Crea et al., 2021; Khandan et al., 2022).

This study critically examines the discrepancies in biomechanical assessments of manual material handling tasks in laboratory versus field settings, focusing on the potential for varied interpretations of ergonomic risks and the effectiveness of assistive devices, including exoskeletons. An experimental approach was adopted by evaluating measurements of muscle activities and body posture, for a manhole cover removal task. This task is a representative high-demand, frequent manual handling activity, commonly performed by utility workers. Experiments were conducted in both lab and field environments, utilizing assistive tools and a passive back-support exoskeleton. Our objectives were to compare biomechanical outcomes in laboratory and field conditions, to evaluate the effectiveness of back support exoskeletons in mitigating ergonomic risks in varied settings, and to contribute to the development of more effective ergonomic assessments and tools in real-world work environments. We hypothesize significant variances in biomechanical assessments between lab and field environments and expect the exoskeleton’s effectiveness in reducing ergonomic risks to differ across these settings. The anticipated results are expected to deepen our understanding of ergonomic risk factors across different environments.

## 2 Methods

### 2.1 Study design and participants

For the in-field assessment, ten able-bodied male workers from drainage and construction (body mass: 73 ± 18 kg, body height: 180 ± 5 cm, age: 33 ± 7 years) volunteered to perform the manhole removal task on their jobsite. The in-lab data was recorded from ten able-bodied participants (6 males, 4 females, body mass: 63 ± 13 kg, body height: 170 ± 7 cm, age: 26 ± 1 years) among university students. The in-lab setup was designed to replicate the tasks performed by workers on the job site. Participants had no clinical history of lower back pain up to 6 months prior to the study, and written consent was collected from the participants after they were informed of the experimental procedures. The study was approved by the research ethics board of the University of Alberta, ID: Pro00109264.

### 2.2 Experimental procedure

**FIGURE 1:**
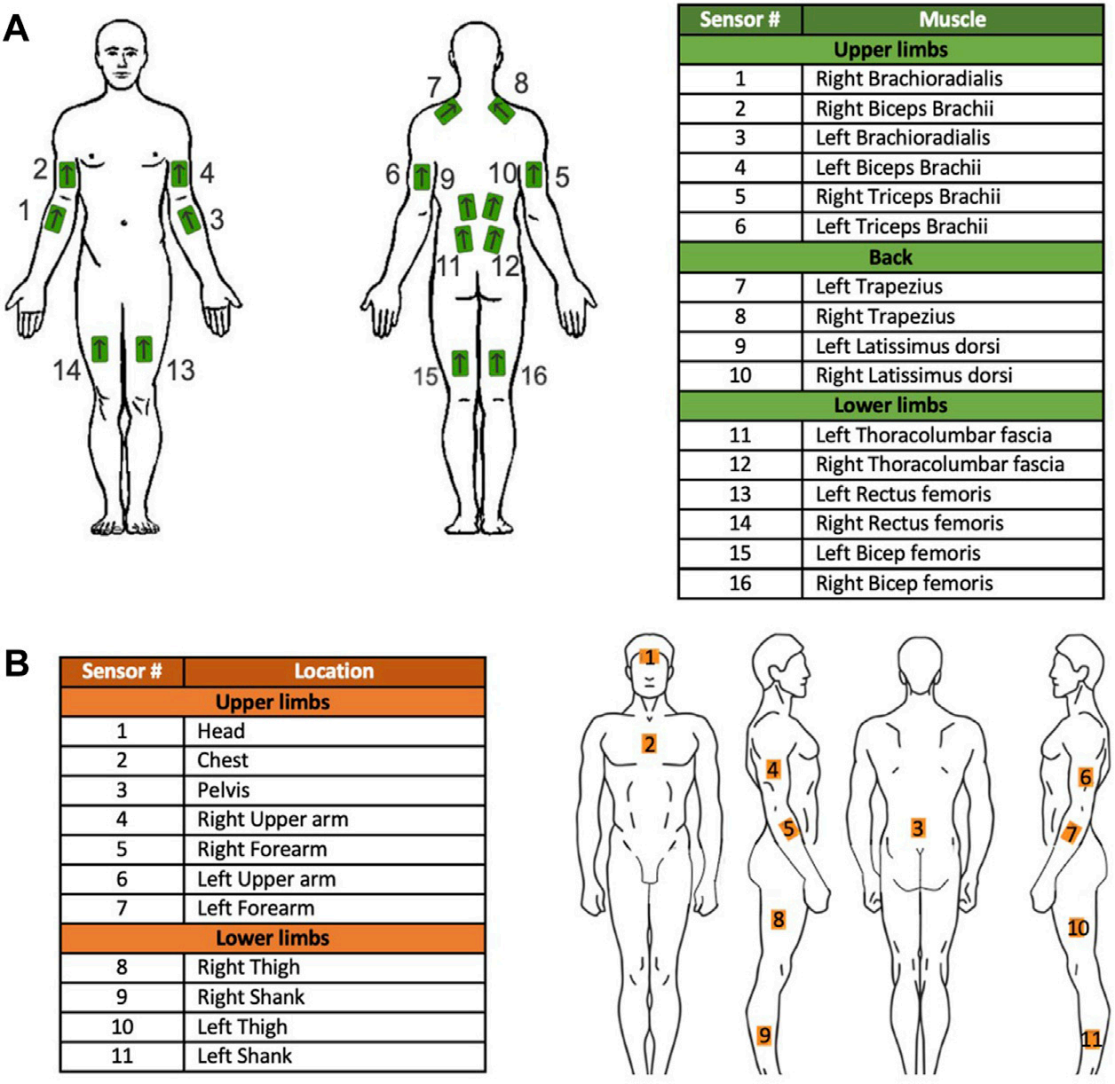
Sensor placement of **(A)** EMG sensors and **(B)** IMU sensors on the participants.

Surface electromyography (EMG) data was captured bilaterally from the Brachioradialis, Biceps Brachii, Triceps Brachii, middle branch of the Trapezius, Latissimus Dorsi, Thoracolumbar Fascia, Rectus Femoris, and Bicep Femoris muscles using 16 Trigno Avanti sensors provided by Delsys Inc., United States (Figure 1). In addition to this, a Rapid Entire Body Assessment (REBA) was conducted to evaluate ergonomic risks, with body joint angles being measured using 11 inertial measurement units (IMUs) from Xsens Technologies, NL. These units were attached to the head, upper trunk (over the sternum), upper arms, forearms, lower back, thighs, and shanks (Figure 1).

**FIGURE 2:**
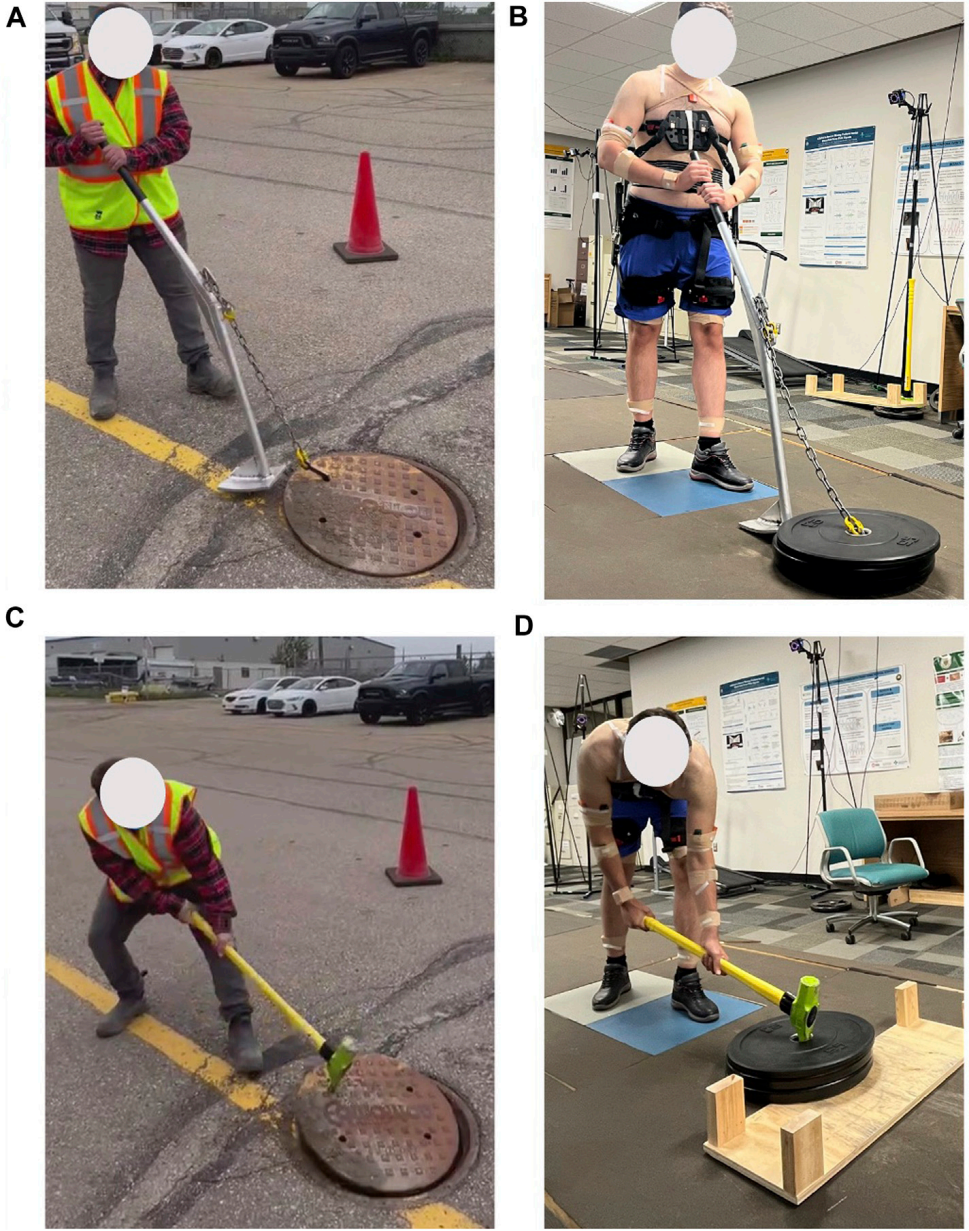
Experimental procedures in-field **(A,C)**, in-lab **(B,D)** using Jake tool **(C,D)**, and Lever tool **(A,B)**.

The activity being assessed involved moving a manhole cover using either a sledgehammer and a pick bar tool called the “Jake” tool or an in-house lever-based tool called the “Lever” tool. Each participant engaged in two repetitions of the task, with a resting period of five seconds of standing still between each repetition, as illustrated in Figure 2. To explore the effectiveness of an exoskeleton in minimizing ergonomic risks, the participants repeated the trials while wearing a passive back-support exoskeleton, the BackX from SuitX, United States. The BackX exoskeleton incorporates passive torque-generating mechanisms around the hip to support the trunk extensor muscles during activities that involve bending and lifting. BackX offers a significant reduction in the load on the lower back, with maximum net torques of 24.8 Nm during flexion, thereby enhancing ergonomic safety (Madinei et al., 2022). Its design features adjustable support settings, including two modes (instant vs. standard) and two levels of support (low vs. high). The instant mode delivers assistive torque immediately upon forward bending, while the standard mode activates supportive torque when trunk flexion reaches approximately 35°. This exoskeleton uses passive mechanisms, including springs, to generate assistive torque only when the user bends forward, without hindering other movements like walking. In the present study, we assessed the exoskeleton’s performance in only instant and its effect on participants’ posture and muscle activities. Each task was performed twice with and without the exoskeleton.

In-field data was collected from utility workers removing manhole covers on the job site. The in-lab trial was designed to duplicate the in-field trials by employing a total of 60 lbs weight plates within the laboratory environment, aiming to replicate the 60 lbs manhole cover used in-field. Furthermore, we maintained consistency by utilizing identical Jake and Lever tools for both the in-field and in-lab tests, ensuring a seamless comparison between the two scenarios. This approach allowed us to assess the performance of the tools and the exoskeleton in a controlled setting, mirroring real-world conditions as accurately as possible. However, there were inconsistencies, such as differences in the experience level between actual workers and student participants. In addition, the in-field manhole covers are flush with the ground which was not possible to exactly replicate in-lab.

### 2.3 Data analysis

The analysis of muscle activities while removing a manhole cover was conducted using EMG sensors. The EMG data was gathered at a rate of 2,000 Hz and then subjected to a band-pass filter, isolating frequencies in the range of 10–500 Hz. Subsequently, the signal was rectified and smoothed using a moving average filter with a window size of 500 data points. In order to standardize the amplitude of the EMG signal, a Maximum Voluntary Contraction (MVC) technique was applied to each of the muscles under observation (Konrad, 2005; Wang et al., 2023). Finally, we determined the Root Mean Square (RMS) value for the normalized amplitude of the EMG signal across the duration of the activity.

The data recorded by IMU, underwent a low-pass filtering process using a 2nd order Butterworth filter set at a 6 Hz cut-off frequency. This filtered data was then used to ascertain the sensor orientations through the application of a sensor fusion algorithm, as described in Nazarahari and Rouhani (2020) and (2021). In addition to this, the orientation of the sensor relative to the body was determined through a functional calibration procedure, as outlined in (Nazarahari et al., 2019). Following these initial steps, the orientations of different body segments were calculated and the angles of various joints during the trials were computed and expressed within the Joint Coordinate System (JCS), as referenced in (Grood and Suntay, 1983).

The calculated joint angles were used to obtain a Rapid Entire Body Assessment (REBA) based on the participants’ body posture and joint angles. REBA is an evaluation tool employed to assess the risk of WMSDs linked to specific job activities. REBA score is used to assess ergonomic risk through the observation of body postures, using the measured joint angle. Each body region is scored separately, and these scores are combined in a two-step table, leading to a single REBA score (Martinez et al., 2022). The accuracy of the REBA score calculated by IMUs was previously validated for manual handling tasks (Humadi et al., 2020; Martinez et al., 2022).

The REBA scores measured using IMU data, and the RMS values of normalized EMG amplitudes for each task were compared between in-field and in-lab experiments. These comparisons were performed with both Lever and Jake tools with and without the exoskeleton. The data did not exhibit a normal distribution, as determined by the Shapiro-Wilk test. Therefore, we chose to employ the Wilcoxon rank-sum test with a significance level of 5% to investigate whether there were any significant differences in the dependent variables among the paired comparisons (Wilcoxon, 1992).

## 3 Results

**FIGURE 3:**
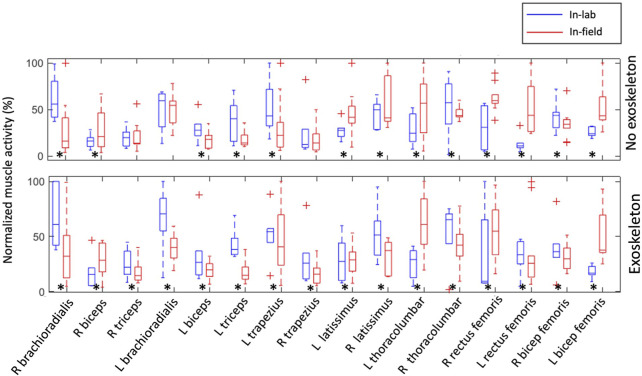
Comparison between normalized muscle activation amplitudes during in-field and in-lab experiments with and without the exoskeleton while using the Jake tool. The results for all participants are presented as boxplots. Crosses indicate an outlier. Black asterisks indicate a significant difference with zero with *p*-values < 0.05.

**FIGURE 4:**
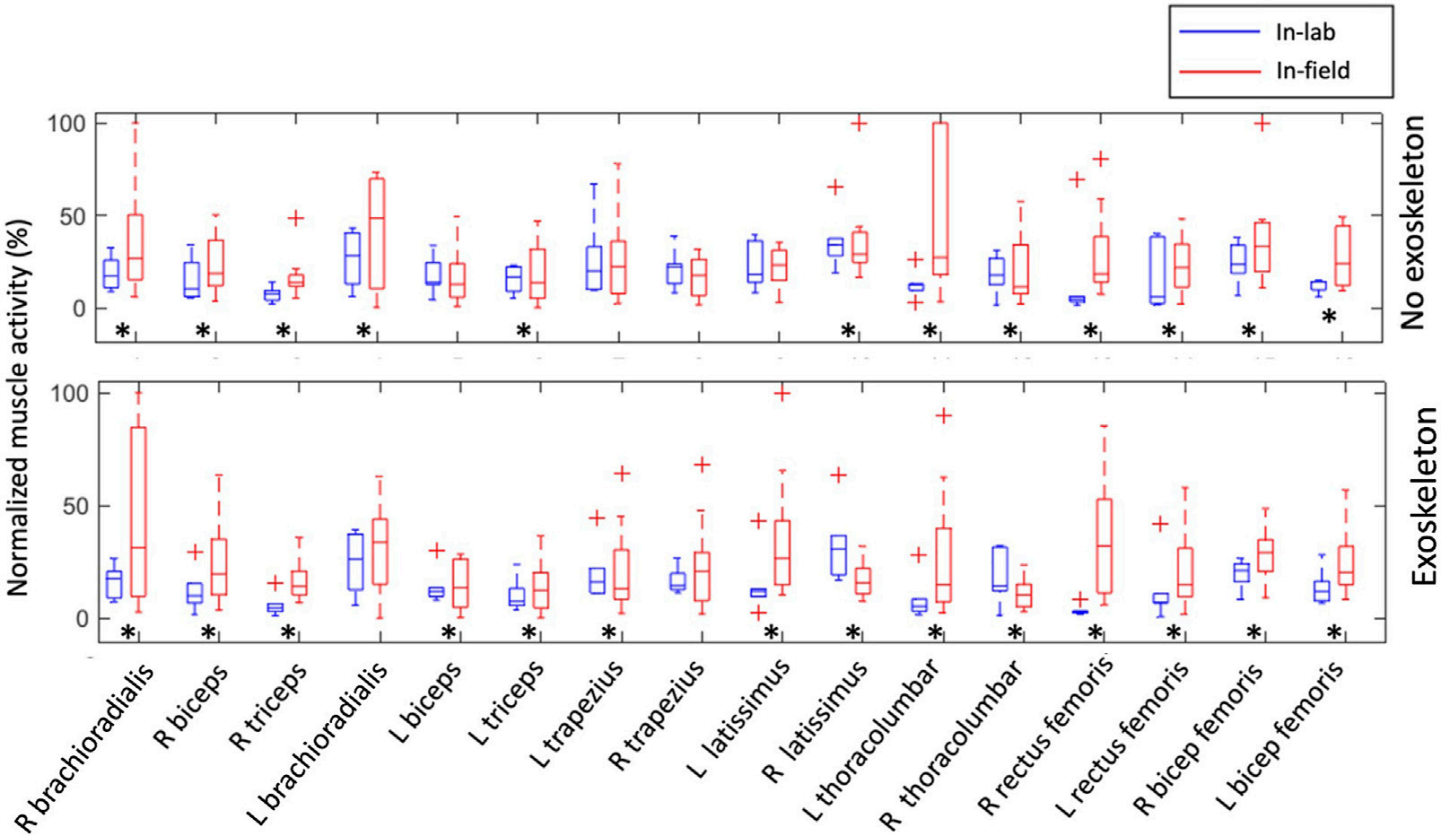
Comparison between normalized muscle activation amplitudes during in-field and in-lab experiments with and without the exoskeleton while using the Lever tool. The results for all participants are presented as boxplots. Crosses indicate an outlier. Black asterisks indicate a significant difference with zero with *p*-values < 0.05.

**FIGURE 5:**
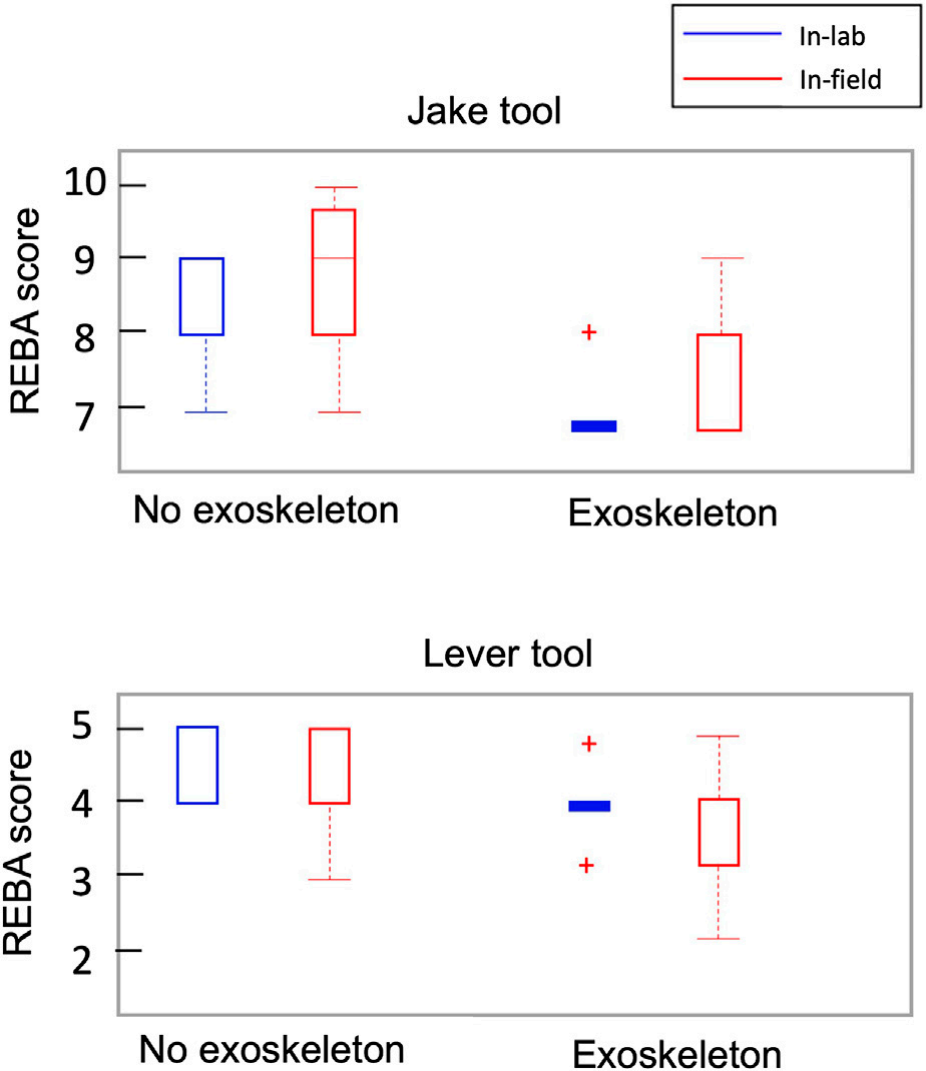
Comparison between REBA scores during in-field and in-lab manhole cover removal experiments using different tools and with and without Exoskeleton. The results for all participants are presented as boxplots. Red crosses indicate an outlier, and there are no significant differences with zero.

The analysis of normalized muscle activity during in-field experiments revealed significant differences (*p* < 0.05) when compared to the in-lab data for most of the muscle groups. Specifically, when participants used the Jake and Lever tools with and without the exoskeleton, the muscle activity levels were notably different in the field compared to the laboratory settings (Figures 3, 4). This suggests that the muscle engagement required for the same task can vary considerably depending on the environment in which the task is performed and participants’ level of experience. Interestingly, despite these differences in muscle activity levels, the REBA scores, which are used to assess posture-related ergonomic risk, showed no statistically significant difference between in-field workers and their in-lab counterparts. This was consistent across scenarios, whether the workers were using the Jake and Lever tools with or without the exoskeleton (Figure 5). This aspect of the results indicates that while the ergonomic posture risk remained consistent across both environments, the actual muscle exertion and patterns of activity differed.

## 4 Discussions

This study aimed to investigate if in-lab experiments with non-workers as participants for ergonomic risk assessment in various tasks and using various tools and exoskeletons can be a reliable surrogate for in-field experiments with actual workers. The findings highlighted that the body postures (assessed by REBA Score) were comparable between the in-lab experiments with non-workers as participants and the in-field experiments with actual experienced workers regardless of the use of exoskeletons or tools. Yet, the muscle activity levels significantly differed between these two conditions showcasing a variance in muscle engagement patterns when tasks were performed with and without the aid of tools and exoskeletons. These differences suggest that the controlled laboratory environment with the use of weights instead of the actual manhole and non-workers as participants instead of actual workers does not fully capture the complexity of real-world tasks, leading to potential discrepancies in ergonomic assessment. This raises intriguing questions about the interpretation of muscle activity data in isolation and highlights the importance of a holistic approach to ergonomic assessment, considering both muscle activity and body posture to gain a comprehensive understanding of the impacts on worker health and safety.

Notably, the observed muscle activities were not higher or lower across all muscle groups in lab experiments compared to the real-world experiments. When using tools and exoskeleton in both modes, the activity level of some muscles increased in lab compared to the real world and decreased for other muscles. This may indicate that actual workers in field employed different muscle recruitment strategy and synergy compared to non-workers in the lab while both participant groups had comparable body postures and performed similar tasks. This might partially be due to demographic differences (such as body height, body mass, age, and sex) between the two participant groups and their different physical fitness and experience level for manual handling task execution. This experience might have led to more efficient movement patterns and muscle use in the field, which were not replicated in the lab setting. Future research should thus aim at deeper understanding of these patterns and their implications for ergonomic interventions. Given that occupational exoskeletons are ultimately intended for use in field environments, it becomes evident that a detailed field analysis with actual workers is essential and likely to yield more insightful results compared to the controlled in-lab studies. In addition, due to the observed different outcomes or exoskeletons among users of different demographics, it is recommended to consider a diverse population in the design and validation of occupational exoskeletons, to ensure that the findings are broadly applicable and inclusive. This diversity should encompass not just gender and age but also physical conditioning and professional experience, as these factors contribute to the efficiency of movement patterns and muscle use.

Besides the difference between the study participants, the differences between experimental conditions can contribute to the observed difference in muscle activities. In a real-world context, numerous uncontrollable variables come into play, such as the varied layers of clothing worn by participants, which can affect movement and muscle engagement. Beyond these, environmental conditions like weather, temperature, and even the time of day may impact the results, which are often unaccounted for in laboratory settings. Moreover, the psychological state of participants, influenced by real-world stressors or the artificial environment of a lab, can also alter performance and outcomes. These discrepancies highlight the need for future studies to perform comprehensive evaluations to understand how each of these factors might influence results differently across various measurement conditions.

In our study, we utilized circular weights with central holes to simulate the lifting of manhole covers, different from manhole covers that often have holes at the edge. In addition, the circular weight were not flush with the ground similar to the real-world manhole cover. This design choice may affect the torque dynamics experienced during actual lifting operations, potentially influencing the ergonomic assessment outcomes. This limitation of our experimental design highlights the importance of designing future studies with closer alignment to real-world conditions to fully understand the ergonomic implications of lifting tasks in utility work.

In this study, we focused on a single material handling task: the removal of utility manhole covers. This task was selected due to its relevance and demand in manual material handling. However, we recognize this as a limitation, as our findings may not fully extend to other types of material handling tasks. Future research could benefit from including a diverse range of scenarios, allowing for a broader understanding of biomechanical and ergonomic impacts across different tasks and enhancing the applicability and generalizability of our findings. In addition, in our study, each participant was tested only twice in each scenario. While this was sufficient to gain preliminary insights, it may impact the overall data reliability and generalizability of the findings. Future studies should consider increasing the number of repetitions to enhance data robustness.

This study focused on comparing the muscle activities and body posture while a back-support exoskeleton was used in a laboratory and in the field. Comparing other factors such as energy consumption (Madinei et al., 2020) between these two experimental conditions should be targeted in the future.

In summary, our study design introduced multiple variables, such as differing environments, experimental setup and participant demographics, which may influence the research outcomes. While this approach provides valuable insights into the real-world application of exoskeletons and tools, it complicates the isolation of single variables to understand their specific impacts. For future research, we recommend controlled studies that isolate and examine an individual factor that may affect the efficacy of exoskeletons and tools in real-world settings. Such studies would complement our findings by providing a deeper understanding of how each variable contributes to the overall effectiveness of tool and exoskeleton interventions in improving worker safety and productivity.

## 5 Conclusion

This study emphasizes the need to evaluate occupational exoskeletons and assistive tools in real-world settings, as muscle activity differs significantly between controlled lab environments and actual field conditions. These insights contribute to a more comprehensive understanding of the practical implications of various tools and exoskeletons employed for physically demanding tasks, such as manhole cover removal and emphasize the importance of considering environmental factors in such ergonomic assessments.

## Data Availability

The raw data supporting the conclusion of this article will be made available by the authors, without undue reservation.
